# Gamete and embryo banks, a necessity or a solution

**Published:** 2013-03

**Authors:** Iman Dezhkam, Hakime Dezhkam, Lotfollah Dezhkam

**Affiliations:** 1 *Student Research Committee, Jahrom University of Medical Sciences, Jahrom, Iran.*; 2 *Young Researchers Club, Jahrom Branch, Islamic Azad University, Jahrom, Iran.*; 3 *Department of Maaref, Jahrom University of Medical Sciences, Jahrom, Iran.*

Dear Editor,

Today some infertile couples who want to have children have request for getting gametes which is a confidential process. According to law, no person must know the identity of the gamete providers (1). Confidentiality of information relating to gamete and embryo donation can cause serious ethical problems. In some countries, forming the Bank of gametes and embryos is intended as a way to prevent privacy problems. The confidentiality of the information may cause several ethical problems that its solution must be looked (2). Therefore we tried to find a new solution for the problem of embryo and gamete Bank which relies on Islamic rules.

In this research, different kind of studies was considered such as relevant books, analytic and review articles applied in different congresses especially on 3^rd^ International Congress of Iranian Society for Reproductive Medicine in the panel of ethics (3, 4).

Some of the most significant reasons expressed for the importance of clarity of donators' information described below: parentage being known, the principle of law and social rights, rule of truth expression, preventing unawareness incest , permanent anxiety for one of the families (donators or receptionists) etc. Accepting security of gamete donation increases the risk of occurring items mentioned above (2, 5). To fix these problems, two solutions have recommended:

1. Creating a Bank of gametes
2. Avoiding security of gamete donation from the beginning, in the other word the necessity of identifying the two families (the donor and the recipient of gametes)

If you create a Bank of gametes, some issues such as incest, is still probable. So before the marriage, those resulted from assisted reproduction, should be introduced to certain centers to guide them not to marry with those who have paternal relationship which is a very important subject in Islam and Muslim nations. In fact according to Islamic rules it is unacceptable even if the probability is very low. In some European countries there is a law that limited the donators not to have more than ten receptionist family (6). So it is better that, children be aware from their biological parents, to be able to deal with this subject conveniently and act wisely. So such people will be avoided being exposed to some realities of their lives which they had not been aware of them until their marriage. The second method emphasizes on recognizing donators and recipients of gametes from the beginning. This will prevent all the probabilities and unwanted effects of security of gamete donation. In most Muslim countries, this solution seems to be more logical because there is not any tolerable reason on the dissimulating the information of two families (the donor and the recipient of gametes).

In present statute in most Islamic countries such as Iran, gamete donation is still secured but there is no reason to continue in this method. So to prevent problems mentioned above and to clarify the paternity of people who are produced from this assisted method, it is recommend avoiding security of gamete donation.

## References

[1] Janssens PMW (2009). Colouring the different phases in gamete and embryo donation. Hum Reprod.

[2] Gong D, Liu YL, Zheng Z, Tian YF, Li Z (2009). An overview on ethical issues about sperm donation. Asian J Androl.

[3] (2002). ESHRE Task Force on Ethics and Law. III. Gamete and embryo donation. Hum Reprod.

[4] (2012). 3rd International and 18th National Congress of Iranian Society for Reproductive Medicine. Iran J Reprod Med.

[5] Larijani B, Zahedi F (2007). Ethical and Religious Aspects of Gamete and Embryo Donation and Legislation in Iran. J Relig Health.

[6] Larijani B, Zahedi F (2007). Ethical Considerations of Gamete and Embryo Donation. Iran J Ethic Sci Tech.

